# Cost-effectiveness of ivosidenib versus chemotherapy for previously treated *IDH1*-mutant advanced intrahepatic cholangiocarcinoma in Taiwan

**DOI:** 10.1186/s12885-024-12362-y

**Published:** 2024-05-22

**Authors:** Kuei-An Chen, Wei-Ming Huang, Eric Yi-Ting Chen, Pei-Kuan Ho, Chen-Han Chueh, Yu-Wen Wen, Ming-Huang Chen, Nai-Jung Chiang, Yi-Wen Tsai

**Affiliations:** 1 Institute of Health and Welfare Policy, National Yang Ming Chiao Tung University, Taipei, Taiwan; 2 Clinical Informatics and Medical Statistics Research Center, Chang Gung University, Taoyuan, Taiwan; 3 Department of Oncology, Taipei Veterans General Hospital, Taipei, Taiwan; 4 School of Medicine, National Yang Ming Chiao Tung University, Taipei, Taiwan

**Keywords:** Ivosidenib, Cost-effectiveness, Advanced intrahepatic cholangiocarcinoma, *IDH1* mutations

## Abstract

**Background:**

International guidelines recommend ivosidenib followed by modified FOLFOX (mFOLFOX) for advanced intrahepatic cholangiocarcinoma (ICC) with isocitrate dehydrogenase 1 (*IDH1*) mutations. Taiwan National Health Insurance covers only fluorouracil/leucovorin (5-FU/LV) chemotherapy for this ICC group, and there has been no prior economic evaluation of ivosidenib. Therefore, we aimed to assess ivosidenib’s cost-effectiveness in previously treated, advanced ICC-presenting *IDH1* mutations compared with mFOLFOX or 5-FU/LV.

**Methods:**

A 3-state partitioned survival model was employed to assess ivosidenib’s cost-effectiveness over a 10-year horizon with a 3% discount rate, setting the willingness-to-pay threshold at 3 times the 2022 GDP per capita. Efficacy data for Ivosidenib, mFOLFOX, and 5-FU/LV were sourced from the ClarIDHy, ABC06, and NIFTY trials, respectively. Ivosidenib’s cost was assumed to be NT$10,402/500 mg. Primary outcomes included incremental cost-effectiveness ratios (ICERs) and net monetary benefit. Deterministic sensitivity analyses (DSA) and probabilistic sensitivity analyses (PSA) were employed to evaluate uncertainty and explore price reduction scenarios.

**Results:**

Ivosidenib exhibited ICERs of NT$6,268,528 and NT$5,670,555 compared with mFOLFOX and 5-FU/LV, respectively, both exceeding the established threshold. PSA revealed that ivosidenib was unlikely to be cost-effective, except when it was reduced to NT$4,161 and NT$5,201/500 mg when compared with mFOLFOX and 5-FU/LV, respectively. DSA underscored the significant influence of ivosidenib’s cost and utility values on estimate uncertainty.

**Conclusions:**

At NT$10,402/500 mg, ivosidenib was not cost-effective for *IDH1*-mutant ICC patients compared with mFOLFOX or 5-FU/LV, indicating that a 50–60% price reduction is necessary for ivosidenib to be cost-effective in this patient group.

**Supplementary Information:**

The online version contains supplementary material available at 10.1186/s12885-024-12362-y.

## Background

Intrahepatic cholangiocarcinoma (ICC), the most prevalent cholangiocarcinoma (CCA) subtype, is a relatively rare cancer, with a higher incidence in Asian populations than in Western countries [1]. The prevalence of isocitrate dehydrogenase 1 (*IDH1*) mutations among patients with ICC ranges from 15 to 20% [2]. Remarkably, approximately 90% of these patients harbor *IDH1* mutations [2]. Both the National Comprehensive Cancer Network (NCCN) and European Society for Medical Oncology (ESMO) guidelines suggest identifying genetic alterations, such as *IDH1* mutations and fibroblast growth factor receptor 2 (*FGFR2*) fusion, as pivotal targets for ICC treatment [3, 4]. Currently, ivosidenib is recommended for the treatment of patients with CCA and *IDH1* mutations who have progressed after receiving at least one prior line of systemic therapy [3, 4].

The phase III ClarIDHy trial assessed the efficacy of ivosidenib (500 mg orally once daily) compared with that of a placebo arm in adult patients with locally advanced or metastatic CCA harboring an *IDH1* mutation following the failure of first-line therapy. The results demonstrated a significant improvement in the primary endpoint of progression-free survival (PFS) in patients treated with ivosidenib compared with that of those who received a placebo (2.7 versus 1.4 months, *p* < 0.001). The crude median overall survival (OS) in the ivosidenib arm did not reach statistical significance compared with that in the placebo arm (10.3 months vs. 7.5 months, *p* = 0.09). This observation was primarily attributed to crossover events among patients originally assigned to the placebo group [5, 6]. However, after adjusting for the crossover effect, the median OS in the placebo group was decreased to 5.1 months, revealing a statistically significant difference in median OS between the two treatment arms (*p* < 0.001) [5, 6]. Based on these positive findings, both the U.S. Food and Drug Administration (FDA) and the European Medicines Agency have approved ivosidenib for the treatment of adult patients with previously treated, locally advanced, or metastatic ICC carrying *IDH1* mutations [7, 8].

Before ivosidenib approval, modified FOLFOX (mFOLFOX), which combines oxaliplatin, folinic acid, and fluorouracil, was the recommended second-line chemotherapy for advanced ICC with *IDH1* mutations as per international guidelines [3, 4]. However, ivosidenib has not been approved by the Taiwan FDA, and mFOLFOX is not fully covered by Taiwan National Health Insurance (NHI) for patients with ICC and *IDH1* mutations. In Taiwan, fluorouracil (5-FU) and leucovorin (LV) regimens are the sole chemotherapy options fully covered by insurance. Given ivosidenib’s high cost, [8, 9] an economic evaluation for reimbursement decisions is necessary, yet no such evaluations have been published. This study aimed to assess ivosidenib cost-effectiveness as a subsequent-line treatment for advanced ICC with *IDH1* mutations in comparison with that of mFOLFOX and 5-FU/LV from the Taiwan National Health Insurance Administration (NHIA)’s perspective and propose a reference price for Taiwan NHIA.

## Methods

### Model structure and overall approach

**Fig. 1:**
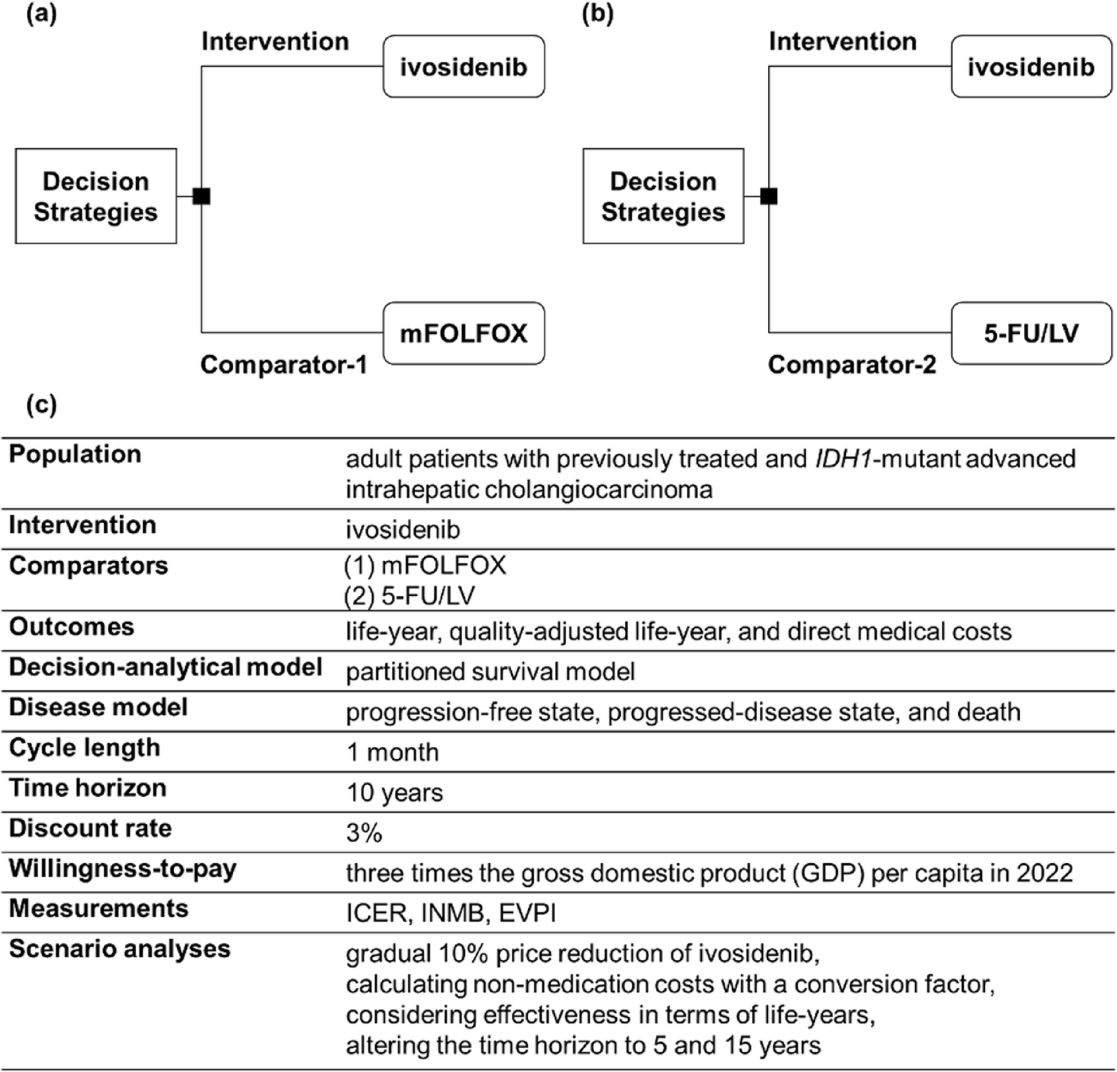
Overall approach of this study. **a** Decision strategies: the intervention regimen represents ivosidenib, while comparator-1 represents mFOLFOX. **b** Decision strategies: the intervention regimen represents ivosidenib, while comparator-2 represents 5-FU/LV. **c** The cost-effectiveness analysis framework. 5-FU/LV, fluorouracil/leucovorin; EVPI, expected value of perfect information; ICER, incremental cost-effectiveness ratio; *IDH1*, isocitrate dehydrogenase 1; INMB, incremental net monetary benefit; mFOLFOX, combination of oxaliplatin, folinic acid, and fluorouracil

To assess ivosidenib cost-effectiveness, its incremental cost and effectiveness were compared using two comparators: (1) comparator-1: mFOLFOX regimen (Fig. 1a), which is recommended by international guidelines and (2) comparator-2: 5-FU/LV regimen (Fig. 1b), which is fully covered by the Taiwan NHI. A partitioned survival model (PSM) [10] was employed, comprising three states: progression-free (PF), post-progression (PP), and death. A 1-month cycle length was employed to assess the cost-effectiveness of ivosidenib over a lifetime, using a ten-year follow-up period. As depicted in Supplementary Fig. 1, until year 10, the overall survival probabilities for both treatment arms were less than one percent, indicating a close approximation to a lifetime follow-up [11]. Health outcomes and direct medical costs are discounted annually at a rate of 3%. The willingness-to-pay (WTP) threshold, as recommended by the World Health Organization [12, 13], was set at 3 times the gross domestic product (GDP) per capita in 2022 (NT$ 2,925,582) [14]. All analyses were conducted from the perspective of Taiwan NHIA. An overview of the cost-effectiveness analysis (CEA) framework is presented in Fig. 1c. This model was structured to align with the natural progression of the disease and was implemented using TreeAge Pro Healthcare (version 2022 R1, Williamstown, MA, USA, TreeAge Software, LLC). This study was reported as per the International Society of Pharmacoeconomics and Outcome Research guidelines and adhered to the consolidated health economic evaluation reporting standards [15, 16].

### Population

This study focused on patients with *IDH1*-mutant advanced ICC. The inclusion criteria and baseline characteristics of participants were similar across the ClarIDHy, ABC-06, [17] and NIFTY trials [18]. The key inclusion criteria in these trials were as follows: being an adult (≥ 18 years old for ClarIDHy and ABC-06; ≥ 19 years old for NIFTY), having documented disease progression after one or two chemotherapy regimens for advanced disease (with no prior IDH1-variant inhibitor therapy in the ClarIDHy trial), and having an Eastern Cooperative Oncology Group (ECOG) performance status score of 0–1 (Additional file 1: Supplementary Table 1).

The median age of the participants was 61 years (range, 33–80) in the ivosidenib arm, 65 years (range, 59–72) in the mFOLFOX arm, and 65 years (range, 37–80) in the 5-FU/LV arm. The proportion of females was similar between the FOLFOX (47%) and 5-FU/LV arms (44%), while it was higher in the ivosidenib arm (65%, Additional file 1: Supplementary Table 2). There is no evidence available to suggest a different ICC prognosis between the sexes [19]; therefore, we assumed that the patients in the ivosidenib arm of the ClarIDHy trial, [5, 6] those in the mFOLFOX arm of the ABC-06 trial, [17] and those in the 5-FU/LV arm of the NIFTY trial [18] were comparable.

### Intervention and comparators

The treatment strategies for both the intervention and comparators in this study were designed as per the established treatment protocols from the three clinical trials. The intervention used ivosidenib, with patients receiving a 500 mg daily dose continuously administered in 28-day cycles, as per the ClarIDHy trial [5, 6]. For comparator-1, the treatment regimen was mFOLFOX. Patients in this group received oxaliplatin at 85 mg/m^2^, L-folinic acid 175 mg (or folinic acid 350 mg), fluorouracil 400 mg/m^2^ (bolus), and fluorouracil 2,400 mg/m^2^ continuous intravenous infusion for 46 h, repeated every 2 weeks, as per the ABC-06 trial [17]. For comparator-2, the treatment regimen was 5-FU/LV, with patients receiving leucovorin 400 mg/m^2^ and fluorouracil 2,400 mg/m^2^ every 2 weeks, following the protocol outlined in the NIFTY trial [18]. We assumed that all treatment strategies would be administered until disease progression, while after disease progression, all patients would receive standardized supportive care in our study.

### Main outcomes

In the CEA, we calculated the incremental cost-effectiveness ratio (ICER) as the incremental cost per incremental quality-adjusted life-year (QALY) associated with ivosidenib in comparison with two different treatment regimens: mFOLFOX (comparator-1) or 5-FU/LV (comparator-2) within this study. Subsequently, we evaluated the resulting ICERs against a WTP threshold of New Taiwan Dollar (NT$) 2,925,582 per QALY. Furthermore, to quantify the net monetary gain attributed to ivosidenib across the two different treatment regimens, we calculated the incremental net monetary benefit (INMB).

### Model inputs

#### Effectiveness

To estimate survival functions, we utilized Webplotdigitizer [20] (version 4.6) to extract discrete data points from the Kaplan–Meier (KM) curves presented in the ClarIDHy, [5, 6] ABC-06, [17] and NIFTY [18] trials. Subsequently, we employed R software (version 4.2.2, Vienna, Austria, R Foundation for Statistical Computing) to generate pseudo-individual data using the algorithm developed by Guyot et al. [21]. We utilized the IPDfromKM [22] package from R software to assess the precision of the OS and PFS plots from published trials and those derived from our reconstructed data points.

Next, we applied six standard parametric models as per the National Institute for Health and Care Excellence (NICE) guidance to fit the reconstructed data points [23]. These models encompassed exponential, Weibull, Gompertz, log-logistic, log-normal, and generalized gamma distributions. The selection of the best-fitting and most appropriate extrapolated survival model was based on a comprehensive evaluation including visual assessment, Akaike information criterion (AIC), Bayesian information criterion (BIC), and expert opinion. We selected the distributions with the lowest AIC and BIC values as the best-fit models for all treatment regimens. Following these assessments, it was determined that all PFS and OS curves for the three treatments followed a log-normal distribution (AIC and BIC values for each distribution are presented in Additional 1: Supplementary Table 3).

Finally, we constructed PFS and OS curves for each arm by combining the KM curves within the trial period with extrapolated parametric models extending beyond the follow-up period, guided by the best-fit survival model (Additional file 2: Supplementary Fig. 1a, 1b, and 1c). Detailed parameters of the survival models are listed in Table 1.

**Table 1:** Model parameters, baseline values, ranges, and distribution for sensitivity analyses

Parameters		Base-case analysis	One-way sensitivity analysis		Probabilistic sensitivity analysis			Source
		Value	Range (± 25%)		Distribution	Parameter 1	Parameter 2	
**Overall survival**								
Ivosidenib:	meanlog	2.2773	2.0715	2.4831	normal (mean, se)	2.2773	0.105	[6]
lognormal	sdlog	1.13	0.9665	1.2935	normal (mean, se)	1.13	0.0834	
mFOLFOX:	meanlog	1.8419	1.6522	2.0316	normal (mean, se)	1.8419	0.0968	[17]
lognormal	sdlog	0.8586	0.7187	0.9985	normal (mean, se)	0.8586	0.0714	
5-FU/LV:	meanlog	1.7813	1.6080	1.9546	normal (mean, se)	1.7813	0.0884	[18]
lognormal	sdlog	0.792	0.6601	0.9239	normal (mean, se)	0.792	0.0673	
Progression-free survival								
ivosidenib:	meanlog	1.199	0.9893	1.4087	normal (mean, se)	1.199	0.107	[5]
lognormal	sdlog	1.049	0.8785	1.2195	normal (mean, se)	1.049	0.087	
mFOLFOX:	meanlog	1.4414	1.2809	1.6019	normal (mean, se)	1.4414	0.0819	[17]
lognormal	sdlog	0.7361	0.6205	0.8517	normal (mean, se)	0.7361	0.059	
5-FU/LV:	meanlog	0.8171	0.5935	1.0407	normal (mean, se)	0.8171	0.1141	[18]
lognormal	sdlog	1.0347	0.8705	1.1989	normal (mean, se)	1.0347	0.0838	
Medication cost (per year, NT$)								
Ivosidenib	Exchange rate between NT and US dollar	29.81			uniform (min, max)	27.93	31.01	[29]
	Medication cost	3,744,552	2,808,414	4,680,690				assumption
mFOLFOX		132,708						NHIA
5-FU/LV		26,124						NHIA
Non-medication cost (per year, NT$)								
Ivosidenib		291,576	218,682	364,470	gamma (α, λ)	16.56	0.00006	[27]
Chemotherapy		856,986	642,740	1,071,233	gamma (α, λ)	59.94	0.00007	[27]
** Supportive care cost (per year, NT$)**		497,710	373,283	622,138	gamma (α, λ)	69.3	0.00014	
**Utility**								
Progression-free state		0.76	0.57	0.95	beta (α, β)	4.7	1.5	[25, 27, 28]
Post-progression state		0.68	0.51	0.85	beta (α, β)	29	13.6	[25, 27, 28]
**Disutility**								
Intravenous (IV) therapy		0.025	0.01875	0.03125				[24, 25, 27, 28]
Grade 3 and higher AE		0.16	0.12	0.2	beta (α, β)	36	193	[26–28]
**Discount rate (per year)**		0.03	0	0.05				
**Conversion factor**		0.9			uniform (min, max)	0.8	1	Assumption

Costs are listed in 2022 New Taiwan dollars

*5-FU/LV *Fluorouracil/leucovorin, *AE *Adverse events, *max *maximum, *mFOLFOX *combination of oxaliplatin, folinic acid, and fluorouracil, *min *minimum, *NHIA *National Health Insurance Administration, *se *standard error, *US *United States

#### Adverse events

We calculated the percentage of grade 3 or higher adverse events within each treatment arm by dividing the number of patients who experienced such events by the total number of patients enrolled in the respective clinical trials. The proportions of grade 3 or higher adverse events within each treatment arm during each treatment cycle were 19.2%, 4.8%, and 1.3% for the ivosidenib, mFOLFOX, and 5-FU/LV arms, respectively. We assumed that adverse events occurred consistently at a uniform rate during each treatment cycle, while the patients were in the PF state.

#### Utilities

In the absence of published utility and disutility data specific to patients with ICC, this study followed NICE’s technology appraisal guidance (TA722 and TA474) [24, 25] and a published study, [26] adopting the utility parameters in patients with advanced hepatocellular carcinoma using sorafenib, as was done in our previous studies on cost-effectiveness in patients with ICC [27, 28]. The assigned utility values for each health state in our model were 0.76 for the PF state and 0.68 for the PP state. Both utility values were assumed to follow a beta distribution. For the same reason, we drew upon disutility values of adverse events from patients with metastatic renal carcinoma, which had been employed in a prior hepatocellular carcinoma study [26]. We set the disutility values for adverse events at 0.16 and subcutaneous or intravenous therapies at 0.025. Similar to utility values, both disutility values were assumed to follow a beta distribution (Table 1) [23, 26].

#### Direct medical cost

The direct medical costs associated with the PF state comprise both medication and non-medication costs, which were reimbursed by the NHIA. The medication costs for the two comparator regimens, mFOLFOX and 5-FU/LV, are based on parameters reported in the literature [27, 28]. Because ivosidenib has not been approved by the Taiwan FDA, there is no market price for it in Taiwan. Therefore, we estimated the hypothesized price for 500 mg of ivosidenib in Taiwan based on the wholesale acquisition cost in the U.S. This cost was adjusted by the ratio of the NHI listing price of pemigatinib in Taiwan to the wholesale acquisition cost of pemigatinib in the U.S. Subsequently, the cost was converted into New Taiwan Dollars, utilizing the average currency exchange rate of 29.81, as reported by Taiwan NHIA between January and March 2023 [29]. This calculation yielded a hypothesized price of NT$10,402 for ivosidenib 500 mg (equivalent to NT$312,046 per year) [9]. To address potential variability, we assumed the exchange rate follows a uniform distribution, ranging from a maximum of 31.01 to a minimum of 27.93, based on data reported by Taiwan NHIA [29] between January 2019 and March 2023.

The non-medication costs associated with PF state were derived from literature [27, 28], encompassing all expenses covered by the NHIA, including diagnostic, medical service, adverse event management, and administrative fees. The non-medication costs for both mFOLFOX and 5-FU/LV were assumed to be identical, and the non-medication costs for the ivosidenib arm were assumed to be the same as those associated with the pemigatinib arm.

The direct medical costs linked to the PP state mainly consist of supportive care expenses covered by the NHIA (NT$37,518 per cycle, Table 1) [27, 28], assumed to be consistent across intervention and comparator arms. We assume treatment-related adverse effects occur only during PFS, and do not extend into the PP state, hence not affecting supportive care costs in our study. For similar reasons, we assume no difference in PP condition between patients receiving ivosidenib and pemigatinib, despite potential variations in treatment-related adverse events during PFS.

All other costs were assumed to follow gamma distributions and were derived from the NHI claims data, as reported in a previous study [27]. Taiwan NHIA employs a point-based fee system, in which the monetary value of each point could be influenced by the conversion factor of the global budget and region, potentially resulting in a monetary value of less than NT$1. To simplify the calculations in the base-case analysis, we assumed that each point is equal to NT$1. In our scenario analysis, we applied a conversion factor of 0.9 (uniform distribution) to non-medication costs to allow for a more flexible assumption (Table 1).

### Uncertainty analyses

To ensure the robustness of our findings and account for uncertainty in the input parameters of the base-case results, we performed both deterministic sensitivity analyses (DSA) and probabilistic sensitivity analyses (PSA) to compare ivosidenib with the two comparator regimens. In the DSA, we varied the value of each parameter individually within their respective 95% confidence intervals. In cases where data were unavailable, we assumed a variance of 25% from the baseline value. For the PSA, we conducted a Monte Carlo simulation involving 1,000 iterations. We generated cost-effectiveness acceptability curves (CEAC) and calculated the expected value of perfect information (EVPI) using predefined distributions for each parameter.

Additionally, scenario analyses were conducted to explore the impacts of various conditions on the results. These scenarios included a gradual 10% price reduction of ivosidenib (ranging from 90 to 40% of the hypothesized price), calculating non-medication costs with a conversion factor, considering effectiveness in terms of life-years, and altering the time horizon to 5–15 years. All parameters in the scenario analyses were applied with the same range or distribution as those used in the base-case analyses, including DSA and PSA, except for the conversion factor parameter, which was set to a 10% variance in the DSA and followed a uniform distribution in the PSA.

### Model validation

We used the Assessment of the Validation Status of Health-Economic decision models to validate our model [30]. Our conceptual model adhered to the approach adopted by the NICE and the Canadian Agency for Drugs and Technologies in Health. However, owing to the lack of individual patient data from pivotal trials, it was not feasible to evaluate differences in mortality between the PF and PP states. Therefore, we could not present a Markov model for direct comparison with the PSM. To ensure the accuracy of our model, three researchers within our group conducted a thorough review of the computer model using TreeAge Pro Healthcare and the associated R programs to identify and rectify any logical errors.

## Results

### Base-case analysis

Compared with mFOLFOX, ivosidenib yielded an incremental gain of 0.37 QALY at an incremental cost of NT$2,346,032. This resulted in an ICER of NT$6,268,528 per QALY and an INMB of NT$ − 1,251,116. In comparison with the 5-FU/LV regimen, ivosidenib provided an incremental benefit of 0.44 QALY, incurring an incremental cost of NT$2,478,942 and resulting in an ICER of NT$5,670,555 per QALY and an associated INMB of NT$ − 1,199,993.

### Base-case sensitivity analysis

**Fig. 2:**
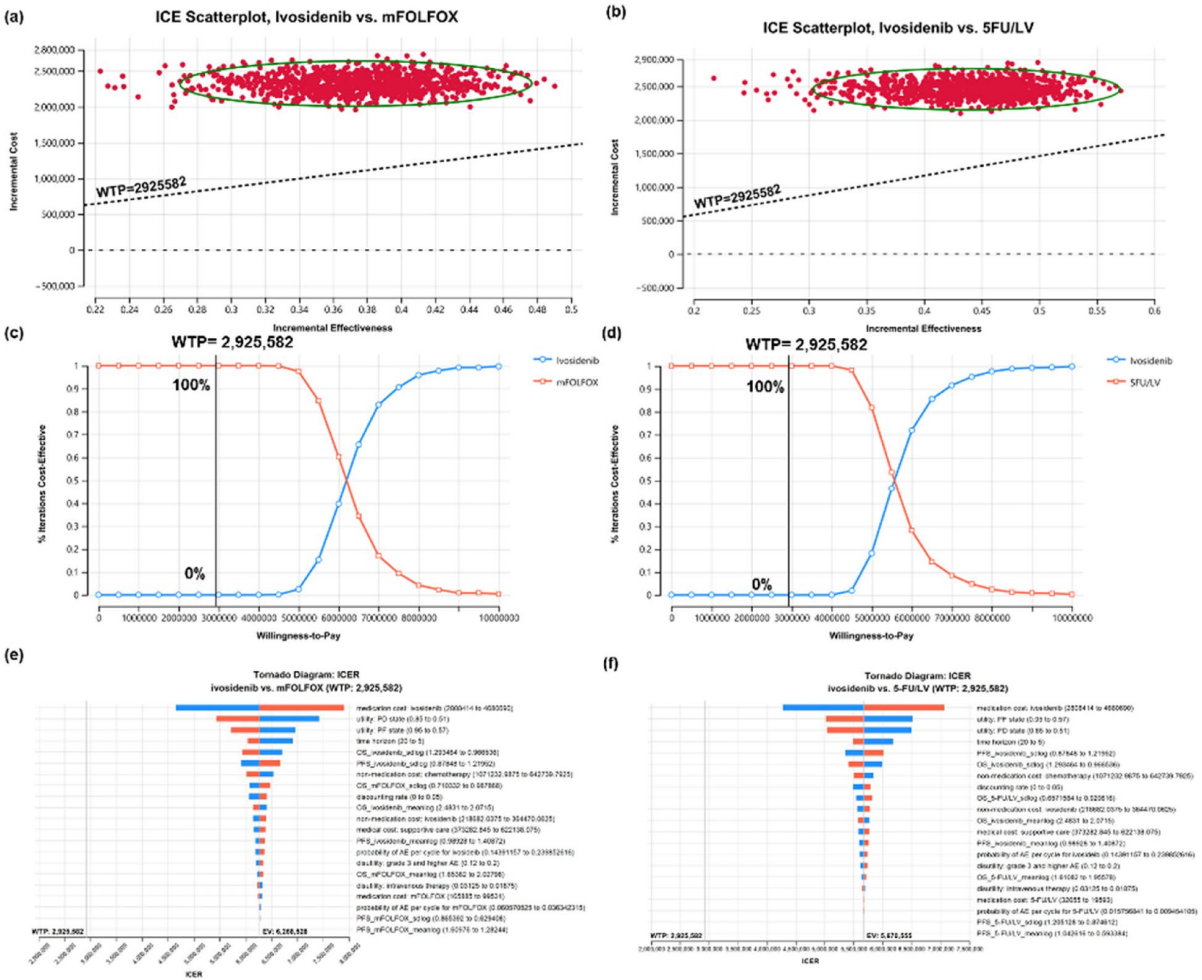
Incremental cost-effectiveness plane for ivosidenib versus mFOLFOX (**a**) and 5-FU/LV (**b**); cost-effectiveness acceptance curve for ivosidenib versus mFOLFOX (**c**) and 5-FU/LV (**d**); and tornado diagram for ivosidenib versus mFOLFOX (**e**) and 5-FU/LV (**f**). Costs are listed in 2022 New Taiwan dollars. The black lines represent a willingness-to-pay threshold of NT$2,925,582 per QALY. 5-FU/LV, fluorouracil/leucovorin; AE, adverse event; EV, expected value; ICE, incremental cost-effectiveness; ICER, incremental cost-effectiveness ratio; mFOLFOX, a combination of oxaliplatin, folinic acid, and fluorouracil; NT, New Taiwan; OS_5FU/LV_meanlog, meanlog parameter of overall survival (5-FU/LV); OS_5FU/LV_sdlog, sdlog parameter of overall survival (5-FU/LV); OS_ivosidenib_meanlog, meanlog parameter of overall survival (ivosidenib); OS_ivosidenib_sdlog, sdlog parameter of overall survival (ivosidenib); OS_mFOLFOX_meanlog, meanlog parameter of overall survival (mFOLFOX); OS_mFOLFOX_sdlog, sdlog parameter of overall survival (mFOLFOX); PD, progressed disease; PF, progression free; PFS_5FU_meanlog, meanlog parameter of progression-free survival (5-FU/LV); PFS_5FU_sdlog, sdlog parameter of progression-free survival (5-FU/LV); PFS_ivosidenib_meanlog, meanlog parameter of progression-free survival (ivosidenib); PFS_ivosidenib_sdlog, sdlog parameter of progression-free survival (ivosidenib); PFS_mFOLFOX_meanlog, meanlog parameter of progression-free survival (mFOLFOX); PFS_mFOLFOX_sdlog, sdlog parameter of progression-free survival (mFOLFOX); QALY, quality-adjusted life-year; WTP, willingness to pay

The cost-effectiveness plane (Fig. 2a and b) illustrates the incremental cost and incremental effectiveness of ivosidenib compared with mFOLFOX and 5-FU/LV, using a Monte Carlo simulation with 1,000 iterations. All 100% of the iterations fell within the first quadrant of the cost-effectiveness plane, signifying that ivosidenib yielded greater effectiveness at higher costs. Under a WTP threshold of 3 times the GDP per capita per QALY gained, ivosidenib seemed unlikely to be cost-effective when compared with both mFOLFOX and 5-FU/LV (Fig. 2c and d). The EVPI for ivosidenib, compared with both mFOLFOX and 5-FU/LV, was NT$0/person (Table 2). DSA revealed that the ICERs for both comparator regimens were most sensitive to variations in the medication cost of ivosidenib and the utilities associated with the PF and PP states (Fig. 2e and f).

**Table 2:** Base-case results

Treatment strategies	Outcomes of Partitioned Survival Models			Incremental Changes	
	Intervention ivosidenib	Comparator-1 mFOLFOX	Comparator-2 5-FU/LV	Ivosidenib vs. mFOLFOX	Ivosidenib vs. 5-FU/LV
Cost					
PF state	2,629,218	453,061	312,157		
Overall	2,945,117	599,085	466,176	2,346,032	2,478,942
LY					
PF state	0.65	0.46	0.35	0.19	0.30
Overall	1.08	0.66	0.56	0.43	0.52
QALYs					
PF state	0.48	0.33	0.26	0.15	0.22
Overall	0.91	0.53	0.47	0.37	0.44
ICER					
Incremental cost per LY gained				5,510,540	4,775,303
Incremental cost per QALY gained				6,268,528	5,670,555
INMB					
LY				− 1,100,508	− 960,222
QALY				− 1,251,116	− 1,199,993
EVPI/person				0	0

Costs are listed in 2022 New Taiwan dollars

*5-FU/LV *Fluorouracil/leucovorin, *EVPI *Expected value of perfect information, *ICER *Incremental cost-effectiveness ratio, *INMB *Incremental net monetary benefit, *LY *Life-year, *mFOLFOX *Combination of oxaliplatin, folinic acid, and fluorouracil, *PF *Progression-free, *QALY *Quality-adjusted life-year

### Scenario analysis

In a scenario analysis of a gradual 10% price reduction for 500 mg of ivosidenib, we found that the price of ivosidenib would need to be reduced by more than 50% for it to become a cost-effective treatment option. Compared with mFOLFOX, a scenario with a 60% reduction (equivalent to 40% of the hypothesized price) resulted in an ICER of NT$2,357,919 per QALY, an INMB of NT$212,451, and a 93.3% probability of cost-effectiveness. Compared with 5-FU/LV, scenario analysis with a 50% reduction yielded an ICER of NT$2,880,642 per QALY, an INMB of NT$19,646, and a 55.6% probability of cost-effectiveness. The CEA results were not sensitive to the remaining scenarios, such as using life years as the outcome measure instead of QALY, alternative conversion factors, and changes in the time horizon (Table 3).

**Table 3:** Scenario analyses

Scenario	Base-case analysis				Probabilistic sensitivity analysis			
	Ivosidenib vs. mFOLFOX		Ivosidenib vs. 5-FU/LV		Ivosidenib vs. mFOLFOX		Ivosidenib vs. 5-FU/LV	
	ICER	INMB	ICER	INMB	Probability of being cost-effective	EVPI/person	Probability of being cost-effective	EVPI/person
Base-case (NT$10,402 per 500 mg)	6,268,528	− 1,251,116	5,670,555	− 1,199,993	0%	0	0%	0
90% price of ivosidenib (NT$9,362 per 500 mg)	5,616,760	− 1,007,188	5,112,572	− 956,065	0%	0	0%	0
80% price of ivosidenib (NT$8,322 per 500 mg)	4,964,992	− 763,260	4,554,590	− 712,137	0%	0	0%	0
70% price of ivosidenib (NT$7,281 per 500 mg)	4,313,223	− 519,332	3,996,607	− 468,209	0%	0	0.2%	135
60% price of ivosidenib (NT$6,241 per 500 mg)	3,661,455	− 275,405	3,438,624	− 224,282	3.5%	1,498	11.6%	6,868
50% price of ivosidenib (NT$5,201 per 500 mg)	3,009,687	− 31,477	2,880,642	19,646	45.9%	220,063	55.6%	185,435
40% price of ivosidenib (NT$4,161 per 500 mg)	2,357,919	212,451	2,322,659	263,574	93.3%	5381.25	92.6%	7577
Applying a conversion factor to non-medication costs	6,322,601	− 1,271,354	5,659,604	− 1,211,292	0%	0	0%	0
Time horizon: 5 years	6,905,934	− 1,100,508	6,178,557	− 1,254,474	0%	0	0%	0
Time horizon: 15 years	6,100,926	− 1,298,030	5,540,330	− 1,178,043	0%	0	0%	0
Life-year as effectiveness	4,385,468	− 780.975	3,977,780	− 655,727	0%	0	0%	0

*5-FU/LV *Fluorouracil/leucovorin, *EVPI *Expected value of perfect information, *ICER *Incremental cost-effectiveness ratio, *INMB *Incremental net monetary benefit,* mFOLFOX *Combination of oxaliplatin, folinic acid, and fluorouracil

## Discussion

### Main findings and policy implications

Our study examined the cost-effectiveness of the ivosidenib regimen based on findings from the ClarIDHy phase III trial among patients with advanced ICC and the *IDH1*-mutant compared with that of mFOLFOX (as per the NCCN and ESMO guidelines) and 5-FU/LV (the only reimbursed regimen by the Taiwan NHI for ICC with *IDH1* mutations). The results showed that the ivosidenib regimen, hypothetically priced at $10,402 per 500 mg, was not cost-effective from the perspective of Taiwan NHIA when compared against the mFOLFOX and 5-FU/LV regimens. Ivosidenib exhibited an ICER of NT$6.3 million when compared with mFOLFOX and NT$5.7 million when compared with 5-FU/LV. The INMB against both comparators was approximately NT$ − 1.2 million. These results strongly suggest that ivosidenib is unlikely to be a cost-effective option when compared with both comparator regimens. A 60% (NT$4,161 per 500 mg) and 50% (NT$5,201 per 500 mg) cost reduction in the hypothesized ivosidenib price would be necessary to lead to cost-effectiveness probabilities of approximately 93.3 and 55.6%, respectively, compared with mFOLFOX and 5-FU/LV.

This study utilized an analytical framework and parameters similar to those used in previous research that assessed pemigatinib cost-effectiveness in patients with advanced ICC and *FGFR2* fusion [27, 28]. A comparison of these CEA findings demonstrated that ivosidenib ICER was notably higher than that of pemigatinib. In addition to the inherent differences in genetic mutations between these two patient populations, the variation in CEA results could be largely attributed to the divergent efficacies observed in the respective pivotal trials. When measured against mFOLFOX and 5-FU/LV, ivosidenib exhibited a favorable outcome in terms of OS (10.3 months vs. 6.2 months and 5.5 months), yet it displayed limited enhancement in PFS (2.7 months vs. 4.0 months and 1.4 months). In contrast, pemigatinib displayed remarkable efficacy in terms of both PFS and OS compared to the two treatment comparators. The disparity in efficacy was underestimated because of differences in the baseline severity of the study populations. In the ClarIDHy trial, the intervention arm for ivosidenib included individuals with less severe conditions than the intervention arm for pemigatinib in the FIGHT-202 trial [27, 28]. The FIGHT-202 trial incorporated a cohort in which 8% of patients had an ECOG score of 2, and 13% of the population were undergoing third-line cancer treatment. In contrast, the ClarIDHy trial enrolled participants with an ECOG score of 0–1, and their treatment history did not necessarily extend to third-line interventions.

Notably, Taiwan NHIA recently introduced a conditional listing policy focused on the reimbursement of emerging cancer medications. Under this novel policy, these cancer drugs are temporarily listed and reimbursed for 2 years, during which real-world effectiveness data are collected. Following the accumulation of a 2-year registry, health technology reassessment based on real-world evidence will be conducted, and reimbursement adjustments considered. This policy was implemented in May 2023, with pemigatinib as the inaugural drug. Pemigatinib was listed with a price of NT$12,500 for 13.5 mg [31], which surpasses our initially recommended price by 40% [27, 28]. Our research outcomes will provide valuable insights to be taken into consideration when contemplating the potential listing and reimbursement of ivosidenib in the future under this policy.

### Study design issues

This study employed an analytical framework similar to that utilized in our previous analysis of pemigatinib cost-effectiveness for patients with advanced ICC and *FGFR2* fusions in comparison with mFOLFOX and 5-FU/LV treatments [30]. Given that the efficacies of pemigatinib and 5-FU/LV were obtained from phase 2 trials, FIGHT-202 and NIFTY, we digitalized the PFS and OS curves of pemigatinib, mFOLFOX, and 5-FU/LV from the respective results of the FIGHT-202, ABC-06, and NIFTY trials. We assumed that the patient populations in all three trials were clinically comparable to reconstruct the survival functions required for the PSM.

In this study, both ClarIDHy (ivosidenib) and ABC-06 (mFOLFOX) were phase 3 randomized controlled trials that included common placebo comparators. Ideally, we would have employed a network meta-analysis (NMA) to estimate the indirect hazard ratio (HR) of ivosidenib versus mFOLFOX if the HRs to the placebo had been reported in both trials. However, ABC-06 did not report an HR for PFS with mFOLFOX. One possible solution would involve employing a digitalization method to estimate the HRs for PFS and OS in both trials, followed by an NMA application. However, it is worth noting that the ClarIDHy trial reported a situation in which 70% of participants initially randomized to the placebo group were crossed over to receive ivosidenib during the trial duration. To address this challenge, the trial adopted an intention-to-treat strategy and utilized the rank-preserving structural failure time (RPSFT) method to reconstruct survival curves. This technique allowed the construction of a survival curve in the placebo group, as though crossovers had not occurred, based on the assumption of a consistent treatment effect for all patients, regardless of treatment initiation timing. Nonetheless, the trial did not present an RPSFT-adjusted PFS curve for the placebo group, which poses a limitation. Consequently, relying solely on the digitalization of the raw PFS curve would not be sufficient to accurately estimate the HR of PFS for ivosidenib to implement the NMA approach. Given this limitation, we were unable to use the NMA approach to estimate the indirect HR of ivosidenib compared with mFOLFOX.

This study has some inherent limitations, including the assumption of the target population, parameter estimation, and model structure. First, owing to limitations in accessing trial data, we employed a digitalization method in conjunction with R programming to reconstruct the survival functions for the three treatment regimens. It is important to acknowledge that this approach hinges on the assumption that the target populations across the three trials are comparable. However, as reported in our study, the ClarIDHy trial included a higher proportion of female participants than the other two comparator trials. There is no evidence suggesting a disparity in the prognosis of ICC among different sex groups [19]; thus, we assumed that the patients in the three arms of the pivotal trials were comparable. Consequently, the survival curves for PFS and OS across the three arms were not expected to display significant sex-related differences, and any such variations in the three trials were not anticipated to significantly affect our results.

Second, the cost parameters, including medication costs for comparators and non-medication supportive care, were estimated using real-world data from the NHI in our previous work [27, 28]. One limitation is that the insurance claim database cannot distinguish self-paying circumstances, potentially resulting in misclassifications between treatment groups. Additionally, the characteristics of the population derived from the NHI claims data used in the analysis may not perfectly align with those of the trial populations due to inherent limitations in the database, such as reliance on diagnostic codes for disease definition and lack of clinical and examination information.

Third, assumptions were made regarding cost parameters. Due to the absence of market prices, the medication cost of ivosidenib was estimated by hypothesizing its price based on the U.S. market and adjusting for a price ratio and exchange rate for conversion. Thus, the conclusions drawn from CEA concerning ivosidenib are conditional and should be interpreted cautiously, though they do not influence recommendations on efficient pricing. As ivosidenib is not covered by NHI, we assumed its non-medication cost to be equivalent to pemigatinib. However, this assumption may not hold true due to disparities in baseline characteristics and genotypes between the patient populations. Additionally, equivalent supportive care costs were assumed for ivosidenib and comparators during the post-progression state, based on the assumptions: (1) supportive care in the PP state is provided after systemic treatment termination in PFS and (2) treatment-related adverse effects occurred solely during the PF state and will not extend into the PP state. Because limited subsequent treatment choices are covered by the NHI in the PP state, these assumptions should not influence the cost estimate of supportive care. Given the current circumstances, we deemed it reasonable to presume equivalent supportive care costs in the PP state regardless of systemic treatments in the PF state.

Fourth, the proportion of adverse events included in the model may differ from that reported in trials owing to the absence of information regarding the timing of adverse events. When reporting on clinical trials, it is customary to count each adverse event in an individual only once, considering the highest grade for that patient.

However, because we were unable to access individual patient data, we opted for a conservative calculation method and assumed that adverse events occurred uniformly across all PF states at a constant rate. Finally, PSMs were used to simulate the efficacy outcomes and costs. This approach may lead to overestimation or underestimation of the extrapolated OS curves by ignoring the information on nonfatal events or treatment effects, respectively [10, 32]. However, without access to individual patient data, exploring model uncertainty using a Markov model was not feasible in this study. To address this limitation, we conducted scenario analyses over 5- and 15-year periods to provide insights into the uncertainty surrounding treatment outcomes.

## Conclusions

In the base-case analysis, ivosidenib, an inhibitor targeting mutant *IDH1*, was not cost-effective at NT$10,402 per 500 mg for previously treated patients with advanced ICC and *IDH1* mutations. However, when the medication cost was reduced to NT$4,161 and NT$5,102 per 500 mg, ivosidenib exhibited a high probability of being cost-effective compared with mFOLFOX and 5-FU/LV. Future studies should focus on health technology reassessments of ivosidenib, using real-world data, if Taiwan’s NHIA reimburses it under the conditional listing policy.

### Supplementary Information

Supplementary Material 1.

Supplementary Material 2.

## Data Availability

All data and software used to construct this model are identified either in the manuscript or in cited source references. The R code that support the findings of this study are available upon request from the corresponding authors.

## Abbreviations

5-FU/LV: Fluorouracil and leucovorin
CCA: Cholangiocarcinoma
DSA: Deterministic sensitivity analysis
ECOG: Eastern Cooperative Oncology Group
ESMO: European Society for Medical Oncology
EVPI: Expected value of perfect information
FDA: Food and Drug Administration
FGFR2: Fibroblast growth factor receptor 2
GDP: Gross domestic product
HR: Hazard ratio
ICC: Intrahepatic cholangiocarcinoma
ICER: Incremental cost-effectiveness ratio
IDH1: Isocitrate dehydrogenase 1
INMB: Incremental net monetary benefit
mFOLFOX: Modified FOLFOX
NCCN: National Comprehensive Cancer Network
NHIA: National Health Insurance Administration
NMA: Network meta-analysis
OS: Overall survival
PF: Progression-free
PFS: Progression-free survival
PP: Post-progression
PSA: Probabilistic sensitivity analysis
QALY: Quality-adjusted life-year
RPSFT: Rank-preserving structural failure time
WTP: Willingness-to-pay
