# Supplementary material for: Cost-effectiveness of ivosidenib versus chemotherapy for previously treated IDH1-mutant advanced intrahepatic cholangiocarcinoma in Taiwan
Source: BMC Cancer. 2024 May 22;24:622. doi: 10.1186/s12885-024-12362-y (PMC11110281; doi:10.1186/s12885-024-12362-y)
Supplement: Supplementary file 1 — Supplementary Material 1. [file 12885_2024_12362_MOESM1_ESM.docx]

**Additional File 1**

Supplementary Table 1: Description of enrollment criteria among four trials: ClarIDHy, ABC-06, NIFTY, and FIGHT-202

|  | | | | | |
| --- | --- | --- | --- | --- | --- |
|  |  | ClarIDHy (ivosidenib) | ABC-06 (mFOLFOX) | NIFTY (5-FU/LV) | FIGHT-202 (pemigatinib) |
| Inclusion | histologically/cytologically | nonresectable or metastatic cholangiocarcinoma  and not eligible for curative resection,  transplantation, or ablative therapies. | nonresectable or recurrent/metastatic cholangiocarcinoma,  gallbladder or  ampullary carcinoma | cholangiocarcinoma  (Documented metastatic disease) | cholangiocarcinoma |
|  | radiology measurement | at least one evaluable and  measurable lesion as defined by RECIST v1.1. | In the ASC plus FOLFOX group, the radiological response was evaluated as per RECIST version 1.1 | at least one evaluable and  measurable lesion as defined by RECIST v1.1. | Radiographically measurable or evaluable disease per RECIST  v1.1. |
|  | age | over 18 years | over 18 years | over 19 years | 18 years or older |
|  | previous treatment | at least 1 and no more than 2 prior systemic regimens inclusive of gemcitabine- or 5-FU-containing regimen  for advanced cholangiocarcinoma. | gemcitabine plus cisplatin as the first-line therapy | gemcitabine plus cisplatin as the first-line therapy (patients who completed the adjuvant therapy and had above 6-month  disease-free period were also eligible) | at least one previous systemic cancer therapy (Previous treatment with selective FGFR inhibitors was not permitted) |
|  | ECOG | 0–1 | 0–1 | 0–1 | 0–2 |
|  | adequate renal function | 1. serum creatinine < 1.5 × ULN  2. creatinine clearance ≧50 mL/min | 1.serum urea and serum creatinine < 1.5 × ULN 2. creatinine clearance ≧30 mL/min | creatinine≦1.5 mg/dL | creatinine clearance > 30  mL/min |
|  | adequate liver function | 1. Gilbert's disease  2. total bilirubin ≦ 2 × ULN  3. AST and ALT ≦ 5 × ULN | 1. total bilirubin < 60 μmol/L  2. ALP, AST, ALT ≦ 5 × ULN | AST, ALT ≦100 IU/L (100 U/L) | total bilirubin < 1.5 × ULN, or ≧ 2.5 × ULN for Gilbert syndrome or a disease involving the liver; AST and ALT ≦ 2.5 × ULN |
|  | adequate hematological function | ANC ≧ 1,500/mm^3^ or 1.5 × 10^9^/L Hb ≧ 8 g/dL platelet count ≧ 100 × 10^9^/L | Hb ≧ 100 g/L, WBC ≧ 3.0 × 10^9^/L,  ANC ≧ 2 × 10^9^/L, platelet count  ≧ 100 × 10^9^/L |  |  |
|  | adequate biliary drainage |  | Adequate biliary drainage, with  no evidence of ongoing infection  (Patients on maintenance antibiotics  are eligible when acute sepsis has resolved). |  |  |
|  | gene status | *IDH1* gene-mutated |  |  | tumor assessment for *FGF/FGFR* gene alteration status |
|  | life expectancy | ≧3 months | > 3 months |  | ≧12 weeks (3 months) |
|  | others | Systemic adjuvant chemotherapy will be considered  a line of treatment if there is documented disease progression during or within 6 months of completing the therapy. |  | adjuvant therapy was the only chemotherapy allowed. | previously treated and clinically stable brain or CNS metastases without corticosteroids for at least 4 weeks (corticosteroids were otherwise allowed without restriction) |
| Exclusion | disease history | Patients with active cardiac disease within 6 months before the start of study treatment were excluded:  1. myocardial infarction  2. unstable angina or stroke  3. active hepatitis B or C viral infections  4. known positive HIV antibody results  5. AIDS-related illness | Patients with following clinical evidence were excluded:  1. brain metastatic disease  2. cardiovascular disease | Patients with following clinical evidence within 6 months before the study were excluded:  1. more than grade 2 gastrointestinal disorder or diarrhea  2. severe arterial  thromboembolic events (i.e., myocardial infarction, unstable angina pectoris, or stroke) | HIV infection, active hepatitis B or C virus infection, abnormal echocardiogram or uncontrolled cardiac disease, current evidence of ectopic mineralization or calcification, clinically significant corneal or retinal disorders confirmed by ophthalmological examination |
|  | prior treatment | 1. received a prior IDH inhibitor.  2. received systemic anticancer therapy or an investigational agent less than 2 weeks before day 1 (washout from previous immune-based anticancer therapy being 4 weeks); had received radiotherapy to metastatic sites of disease  less than 2 weeks before day 1; or had undergone hepatic irradiation, chemoembolization, and radiofrequency ablation less than 4 weeks before day 1. | any other form of first-line systemic  chemotherapy (including rechallenge with cisplatin and gemcitabine) was not allowed. |  | selective FGFR inhibitor |

5-FU, fluorouracil; 5-FU/LV, fluorouracil/leucovorin; AIDS, Acquired Immunodeficiency Syndrome; ALP, alkaline phosphatase; ALT, alanine aminotransferase; ANC, absolute neutrophil count; ASC, active symptom control; AST, alanine aminotransferase; CNS, central nervous system; ECOG, eastern cooperative oncology group; FGF, fibroblast growth factor; FGFR, fibroblast growth factor receptor; Hb, hemoglobin; HIV, human immunodeficiency virus; IDH, isocitrate dehydrogenase; *IDH1*, isocitrate dehydrogenase 1; mFOLFOX, combination of oxaliplatin, folinic acid, and fluorouracil; RECIST, Response Evaluation Criteria in Solid Tumors; ULN, upper limit of normal; WBC, white blood cell

| Supplementary Table 2: Description of the four trials, including study populations and trial protocols. | | | | |
| --- | --- | --- | --- | --- |
| Trial | ClarIDHy | ABC-06 | NIFTY | FIGHT-202 |
| Country | France, Italy, South Korea, Spain, United Kingdom, and United States | United Kingdom | South Korea | Belgium, France, Germany, Israel, Italy, Japan, Korea, Spain, Taiwan, Thailand, United Kingdom, United States |
| Population | unresectable or metastatic cholangiocarcinoma with *IDH1* mutation | locally advanced or metastatic biliary tract cancer, including cholangiocarcinoma, gallbladder carcinoma, and ampullary carcinoma | metastatic biliary tract cancer, including intrahepatic and extrahepatic cholangiocarcinoma and gallbladder cancer | locally advanced or metastatic cholangiocarcinoma with and without *FGFR2* fusions or rearrangements |
| Age | median: 61 (33–80) | median: 65 (59–72) | median: 65 (37–80) | median: 56 (26–77) < 65: 77%, 65–74: 19%, ≥ 75: 5% |
| Sex | female: 65% | female: 47% | female: 44% | female: 61% |
| First-line therapy | gemcitabine or a fluorouracil-based chemotherapy regimen, and received no prior IDH-variant inhibitor therapy | gemcitabine + cisplatin | gemcitabine + cisplatin | systemic cancer therapy (previous treatment with selective FGFR inhibitors was not permitted) |
| Intervention | ivosidenib | mFOLFOX | combination of irinotecan, fluorouracil (5-FU), and leucovorin (LV) | pemigatinib |
| Comparator | placebo | active symptom control (ASC) | combination of 5-FU and LV |  |
| Outcome measurement | primary outcome: PFS secondary outcome: OS, ORR | primary outcome: OS secondary outcome: PFS, response rate | primary outcome: PFS secondary outcome: OS, ORR | primary outcome: ORR secondary outcomes: OS, PFS |
| Enrollment (n) | 187 (126 are in the ivosidenib group) | 162 (81 patients are in the mFOLFOX group) | 178 (86 patients are in the 5-FU/LV group) | 147 (107 patients are *FGFR2* fusions/rearrangements) |
| Study type | phase 3, double-blind, RCT | phase 3, open-label, RCT | phase 2b, open-label, RCT | phase 2, open label, single arm |
| 5-FU/LV, fluorouracil/leucovorin; FGFR, Fibroblast growth factor receptor; *IDH1*, isocitrate dehydrogenase 1; mFOLFOX, combination of oxaliplatin, folinic acid, and fluorouracil; ORR, objective response rate; OS, overall survival; PFS, progression-free survival; RCT, randomized controlled trial | | | | |

| Supplementary Table 3: AIC and BIC values from each survival model | | | | | |
| --- | --- | --- | --- | --- | --- |
| Regimen | Distribution | Progression-free survival | | Overall survival | |
|  |  | AIC | BIC | AIC | BIC |
| ivosidenib | Exponential | 417.8749 | 420.6952 | 738.2346 | 741.0709 |
|  | Weibull | 419.7771 | 425.4177 | 738.4342 | 744.1068 |
|  | Log-normal | 395.4979 | 401.1385 | 735.8345 | 741.5071 |
|  | Log-logistic | 399.51 | 405.1505 | 736.4001 | 742.0727 |
|  | Gompertz | 414.946 | 420.5866 | 740.0959 | 745.7684 |
|  | Generalized gamma | 400.709 | 409.1699 | 736.8406 | 745.3495 |
|  | Gamma | 418.3237 | 423.9642 | 737.5566 | 743.2292 |
| mFOLFOX | Exponential | 430.4811 | 432.8756 | 473.2572 | 475.6516 |
|  | Weibull | 417.7545 | 422.5434 | 467.2522 | 472.0411 |
|  | Log-normal | 406.3574 | 411.1463 | 460.9009 | 465.6898 |
|  | Log-logistic | 409.0835 | 413.8724 | 463.7612 | 468.5501 |
|  | Gompertz | 427.3057 | 432.0946 | 472.7546 | 477.5435 |
|  | Generalized gamma | 409.3149 | 416.4983 | 463.1412 | 470.3246 |
|  | Gamma | 412.9695 | 417.7584 | 464.763 | 469.5519 |
| 5-FU/LV | Exponential | 370.2497 | 372.7041 | 448.8268 | 451.2811 |
|  | Weibull | 371.5888 | 376.4974 | 438.1803 | 443.089 |
|  | Log-normal | 349.1462 | 354.0549 | 429.3484 | 434.2571 |
|  | Log-logistic | 353.065 | 357.9737 | 430.816 | 435.7247 |
|  | Gompertz | 368.6886 | 373.5973 | 446.3001 | 451.2088 |
|  | Generalized gamma | 354.2208 | 361.5839 | 431.9701 | 439.3331 |
|  | Gamma | 372.247 | 377.1557 | 434.4464 | 439.3551 |

5-FU/LV, fluorouracil/leucovorin; AIC, Akaike information criterion; BIC, Bayesian information criterion; mFOLFOX, combination of oxaliplatin, folinic acid, and fluorouracil
