# Supplementary material for: Cost-effectiveness of ivosidenib versus chemotherapy for previously treated IDH1-mutant advanced intrahepatic cholangiocarcinoma in Taiwan
Source: BMC Cancer. 2024 May 22;24:622. doi: 10.1186/s12885-024-12362-y (PMC11110281; doi:10.1186/s12885-024-12362-y)
Supplement: Supplementary file 2 — Supplementary Material 2. [file 12885_2024_12362_MOESM2_ESM.docx]

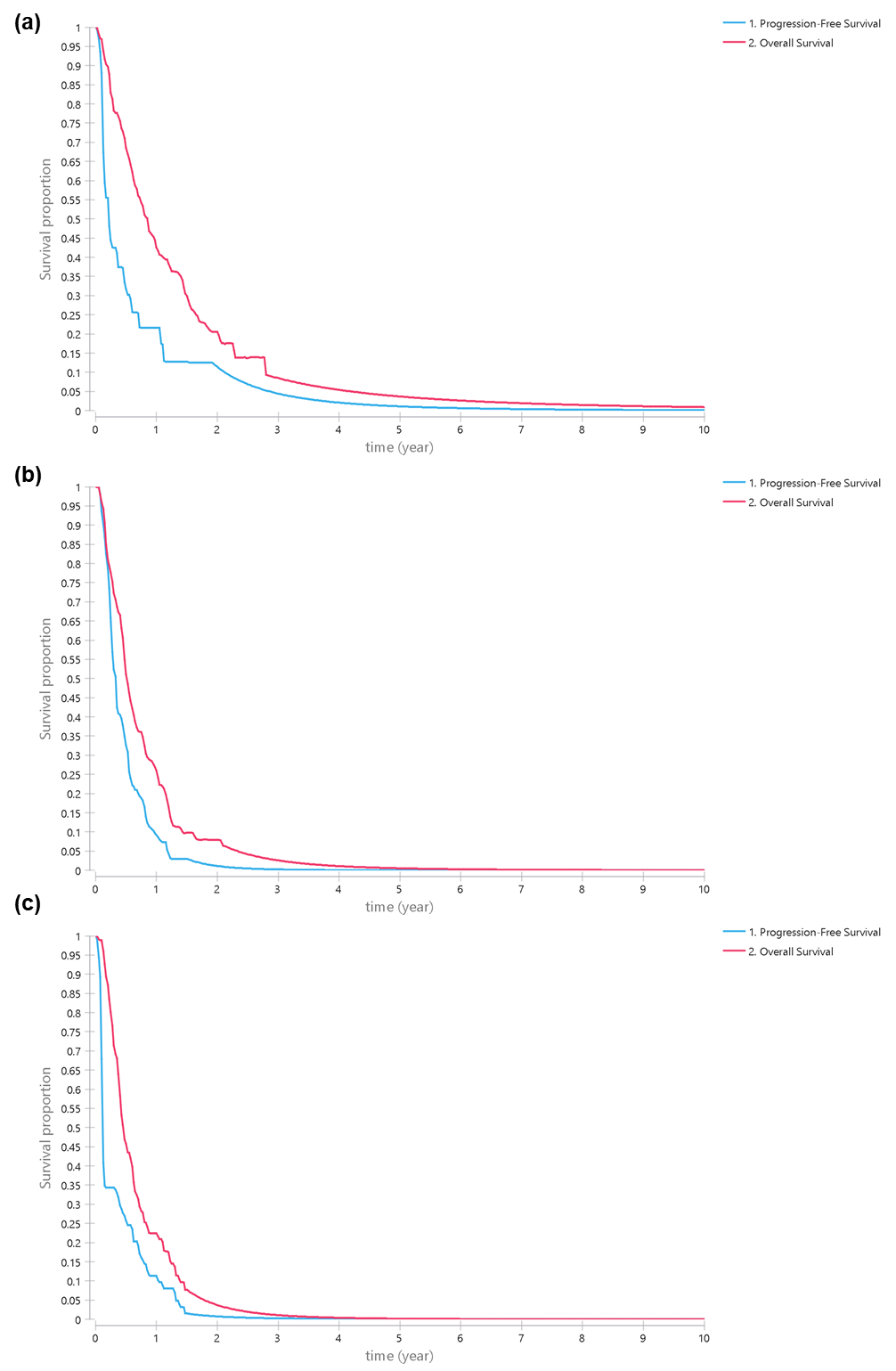


**Supplementary Figure 1:** Hybrid survival curves for (a) ivosidenib, (b) mFOLFOX, and (c) 5-FU/LV were reconstructed using Kaplan–Meier plots and extrapolated using a lognormal distribution. 5-FU/LV, fluorouracil/leucovorin; mFOLFOX, combination of oxaliplatin, folinic acid, and fluorouracil
